# Supplementary material for: Iterative improvement in the automatic modular design of robot swarms
Source: PeerJ Comput Sci. 2020 Dec 7;6:e322. doi: 10.7717/peerj-cs.322 (PMC7924708; doi:10.7717/peerj-cs.322)
Supplement: Supplemental Information 3 [file peerj-cs-06-322-s003.zip › argos3/doc/api/standalone/a00516_source.html]

ARGoS: plugins/robots/generic/control\_interface/ci\_range\_and\_bearing\_sensor.cpp Source File


- Main Page
- Related Pages
- Namespaces
- Classes
- Files

- File List
- File Members

# plugins/robots/generic/control\_interface/ci\_range\_and\_bearing\_sensor.cpp

Go to the documentation of this file.

```
00001 
00007 #include "ci_range_and_bearing_sensor.h"
00008 
00009 #ifdef ARGOS_WITH_LUA
00010 #include <argos3/core/wrappers/lua/lua_utility.h>
00011 #endif
00012 
00013 namespace argos {
00014 
00015    /****************************************/
00016    /****************************************/
00017 
00018    const CCI_RangeAndBearingSensor::TReadings& CCI_RangeAndBearingSensor::GetReadings() const {
00019      return m_tReadings;
00020    }
00021 
00022    /****************************************/
00023    /****************************************/
00024 
00025 #ifdef ARGOS_WITH_LUA
00026    void CCI_RangeAndBearingSensor::CreateLuaState(lua_State* pt_lua_state) {
00027       CLuaUtility::OpenRobotStateTable(pt_lua_state, "range_and_bearing");
00028       for(size_t i = 0; i < m_tReadings.size(); ++i) {
00029          CLuaUtility::StartTable(pt_lua_state, i+1);
00030          CLuaUtility::AddToTable(pt_lua_state, "range", m_tReadings[i].Range);
00031          CLuaUtility::AddToTable(pt_lua_state, "horizontal_bearing", m_tReadings[i].HorizontalBearing);
00032          CLuaUtility::AddToTable(pt_lua_state, "vertical_bearing", m_tReadings[i].VerticalBearing);
00033          CLuaUtility::StartTable(pt_lua_state, "data");
00034          const CByteArray& cData = m_tReadings[i].Data;
00035          for(size_t j = 0; j < cData.Size(); ++j) {
00036             CLuaUtility::AddToTable(pt_lua_state, j+1, cData[j]);
00037          }
00038          CLuaUtility::EndTable(pt_lua_state);
00039          CLuaUtility::EndTable(pt_lua_state);
00040       }
00041       CLuaUtility::CloseRobotStateTable(pt_lua_state);
00042    }
00043 #endif
00044 
00045    /****************************************/
00046    /****************************************/
00047 
00048 #ifdef ARGOS_WITH_LUA
00049    void CCI_RangeAndBearingSensor::ReadingsToLuaState(lua_State* pt_lua_state) {
00050       lua_getfield(pt_lua_state, -1, "range_and_bearing");
00051       /* Save the number of elements in the RAB table */
00052       size_t unLastMsgNum = lua_rawlen(pt_lua_state, -1);
00053       /* Overwrite the table with the new messages */
00054       for(size_t i = 0; i < m_tReadings.size(); ++i) {
00055          CLuaUtility::StartTable(pt_lua_state, i+1);
00056          CLuaUtility::AddToTable(pt_lua_state, "range", m_tReadings[i].Range);
00057          CLuaUtility::AddToTable(pt_lua_state, "horizontal_bearing", m_tReadings[i].HorizontalBearing);
00058          CLuaUtility::AddToTable(pt_lua_state, "vertical_bearing", m_tReadings[i].VerticalBearing);
00059          CLuaUtility::StartTable(pt_lua_state, "data");
00060          const CByteArray& cData = m_tReadings[i].Data;
00061          for(size_t j = 0; j < cData.Size(); ++j) {
00062             CLuaUtility::AddToTable(pt_lua_state, j+1, cData[j]);
00063          }
00064          CLuaUtility::EndTable(pt_lua_state);
00065          CLuaUtility::EndTable(pt_lua_state);
00066       }
00067       /* Are the new messages less than the old ones? */
00068       if(m_tReadings.size() < unLastMsgNum) {
00069          /* Yes, set to nil all the extra entries */
00070          for(size_t i = m_tReadings.size()+1; i <= unLastMsgNum; ++i) {
00071             lua_pushnumber(pt_lua_state,  i);
00072             lua_pushnil   (pt_lua_state    );
00073             lua_settable  (pt_lua_state, -3);
00074          }
00075       }
00076       lua_pop(pt_lua_state, 1);
00077    }
00078 #endif
00079 
00080    /****************************************/
00081    /****************************************/
00082 
00083    CCI_RangeAndBearingSensor::SPacket::SPacket() :
00084       Range(0.0f) {}
00085 
00086    /****************************************/
00087    /****************************************/
00088 
00089 }
```

---

Generated on 10 Jul 2018 for ARGoS by 
 1.6.1
